# Dermatologic Research in Displaced Populations: Importance, Challenges, and Proposed Solutions

**DOI:** 10.2196/64828

**Published:** 2025-05-05

**Authors:** Derek Maas, Jackleen S Marji

**Affiliations:** 1 New York University Grossman School of Medicine, New York, NY, United States; 2 The Ronald O. Perelman Department of Dermatology, New York University Grossman School of Medicine, 335 West 52nd St, 6th Floor, New York, NY, 10016, United States, 1 646-754-2100

**Keywords:** displaced persons, dermatology, dermatologic research, refugees, internally displaced persons, asylum seekers, skin diseases, epidemiology, health services accessibility, trauma-informed care, communicable diseases, vaccination, telemedicine, mental health services, posttraumatic stress disorder, ethical research, health care disparities, global health, infectious, scabies, fungal infections, bacterial infections, war-related injuries, health policy, health care delivery, scars, genital diseases, mental health, research design, cultural competency, informed consent

## Abstract

Displaced populations face complex dermatologic challenges. Contributing factors include low immunization rates, poor sanitation, crowded living conditions, and physical abuse. Chronic inflammatory conditions and infectious diseases, including fungal infections and scabies, are prevalent. Research is crucial to reduce the spread of disease, improve care in these populations, and develop sustainable frameworks for long-term dermatologic health care in crisis settings. The paucity of dermatologist support in this field exacerbates the issue. Ethical considerations include nonmaleficence and culturally sensitive practices, and proposed solutions include trauma-informed care training, advocacy for equitable research funding, teledermatology, and the development of shared international screening guidelines. Further research is essential to enhance dermatologic care for displaced populations.

## Background

For the purpose of this article, the term “displaced person” refers specifically to individuals who have been forcibly displaced due to conflict, persecution, violence, or disasters, including refugees, internally displaced persons, and asylum seekers. The global displacement crisis has led to the forced migration of 122.6 million individuals as of 2024, which increased from 59.2 million in 2014 [1]. Of these individuals, 71% are hosted in low- to middle-income countries, and 40% are children, many of whom encounter significant dermatologic health issues [1,2]. Displaced populations have complex health care needs. Dermatologic conditions, not frequently prioritized in acute care settings, represent significant disease burden and often serve as visible markers of hygiene-related issues, systemic illness, or infectious outbreaks [3]. Infectious diseases such as malaria, measles, acute respiratory infections, and diarrheal illnesses are among the major causes of morbidity and mortality [4]. Along with malnutrition (particularly in children), these problems account for the majority of deaths among displaced persons [4]. Mental health disorders are also prevalent due to the severe psychological stress associated with displacement [5]. This complex interplay of organ systems and the transient nature of the communities pose challenges to conducting dermatologic research, but such targeted research is crucial for understanding and addressing the skin care needs of these individuals [6]. Without it, health care providers lack the necessary epidemiologic data to design interventions and allocate resources effectively. Dermatologists are needed to assist in developing tailored management strategies [2]. We propose practical solutions to improve the mutual benefit of this research.

## Dermatologic Conditions in Displaced Populations

A review of skin diseases in displaced populations [3] notes an increased frequency of cutaneous conditions in the scarce literature available, reporting a prevalence between 18.7% and 96.2% [7-10]. High rates of cutaneous conditions are due to several factors. War and conflict severely damage health care infrastructure [2]. Of surveyed respondents in the Syrian conflict zone, an endemic area of leishmaniasis, 12% knew that they could access treatment at hospitals or health centers, and less than a quarter had heard of the disease’s vector, the tsetse fly [11]. These findings helped drive educational initiatives in the community [11]. This is one example of dermatologists and infectious disease specialists collaborating to guide targeted education to at-risk populations and front-line providers.

Chronic inflammatory conditions are prevalent and often overlooked in displaced populations. Four studies [7-9,12] aligned with this paper’s definition of displaced persons. Migrants in Maltese reception centers (n=2216) had rates of “contact dermatitis and other eczema” of 4.8% [7]. A Jordanian refugee camp study (n=288) reported “dermatitis/eczema” at rates of 33.8%, while a study of migrants on the Mediterranean coast in Italy (n=6188) found rates of dermatitis at 7.5% [8,9]. Further, a diagnosis in the category “dermatitis and eczema” encompassed 21.3% of 380 Rohingya refugees living in the Kutupalong camp in Bangladesh [12]. Other forms of chronic dermatologic conditions have rarely been differentiated in the literature, but the Jordanian and Rohingya population studies specifically identified “papulosquamous disorders,” occurring at rates of 6.9% and 2.9% [8,12]. Research on the management of these conditions in the setting of displacement is a potential area for growth. For example, comparing the effectiveness of various forms of barrier repair could guide the improved preparation of front-line health care providers.

Furthermore, informal settlements of displaced persons include diverse subpopulations with varying immunization levels; the seroprevalence often does not reach levels that confer herd immunity [2]. This, combined with poor sanitation and crowded housing conditions, leads to the rapid spread of contagious and vaccine-preventable diseases like measles and varicella [2,3]. Prior studies have found infectious diseases to represent 20.8% to 72% of skin conditions in displaced persons [7-9,12]. However, rates vary depending on classification criteria and potential diagnostic overlap. Viral infection rates fall between 0.7% and 8.5%, while fungal and bacterial infections have been reported from 7.9% to 49% and 3.2% to 11.2%, respectively [7-9,12]. Understanding local rates of communicable conditions is crucial to developing targeted vaccination efforts.

The process of displacement itself often forces migrants into extreme conditions, with many forced to travel in small boats [2,3]. A common location of arrival for these vessels is south of the Mediterranean Sea, where skin diseases commonly seen include secondary bacterial infections, scabies, deep abscesses, and tissue necrosis [3]. It is well established that scabies is particularly pervasive, with rates ranging from 3.5% to 58% [7-9,12]. A 2007 review studied the efficacy of various scabies treatments in refugee camps, highlighting the success of mass ivermectin administration [13]. The study demonstrated the importance of research in developing effective community interventions.

Current findings show wide variation in the rates of skin manifestations, and more research is needed for effective treatment and prevention. Future studies should further delineate rates of communicable infections by region while spreading the focus to chronic inflammatory conditions.

## Challenges and Ethical Considerations

The backbone of research is ethical practice, and important aspects include nonmaleficence, beneficence, justice, and respect for persons. However, these mainstays are often not adequate for the complexities of vulnerable populations [6]. Access is fraught with difficulties due to safety concerns; many displaced persons lack the legal right to work or reside in their host country and consequently are transiently located with increased risk of arrest and detention [1,6]. For these reasons, individuals may show reluctance to engage in research and be concerned with confidentiality. Furthermore, participants often lean on researchers as a form of support, leading to conflicts of interest and trouble with the informed consent process, which may already be difficult to understand [6]. These considerations underscore the integration of culturally sensitive practices that foster an environment of open communication with balanced power dynamics [2,6]. Clinicians might be hesitant to study these populations in the first place due to anticipated difficulties in securing research approval and funding, given the inequitable distribution of academic funding and resources toward high-income countries [14]. Finally, geopolitical instability further complicates research efforts, as ongoing conflict and the displacement of health care workers hinder the implementation of structured studies; the politicization of global health and power imbalances in research partnerships only serves to exacerbate this challenge [15].

Additional barriers to research specifically apply to dermatology. Notably, screening guidelines for skin diseases vary internationally; the lack of shared guidelines poses a challenge to the design of systematic research on cutaneous conditions and the consistent provision of care [2,3]. It is also important to consider that the process of forced displacement often involves physical abuse and torture [2]. Scars, ecchymoses, and genital lesions are associated with trauma and are seen at high rates in displaced populations [2]. While investigators have infrequently distinguished conditions associated with trauma, scarring was found in migrants living in Maltese reception centers at a rate of 9.5% [3]. The spotlight that dermatologic research can place on cutaneous conditions has the potential to be a trigger that could retraumatize study participants, leading to posttraumatic stress disorder and other adverse mental health conditions [2].

## Proposed Solutions

The risk of retraumatization in displaced populations makes nonmaleficence an ethical consideration of utmost importance [6]. To minimize the potential for psychological harm, clinicians working with these groups should be trained in trauma-informed care. Trauma-informed care training teaches the recognition of actions that could trigger memories of past traumatic events or add new traumatic experiences and requires that clinicians overcome the time constraint barrier of working in humanitarian settings (Table 1) [16]. A facet of this training involves understanding how and where to access mental health resources, which may be difficult during displacement [16]. The use of trauma-informed practices is of particular importance when it comes to dermatologic conditions because of their visibility and frequent direct association with physical trauma.

**Table 1.** Proposed solutions and their potential impacts on improving dermatologic research with displaced populations.

Proposed solution	Potential impact	Explanation	Major obstacles
Teledermatology	↑ Continuity of research ↓ Spread of disease	Telemedicine platforms reduce the transmission of infections, provide consistent access to dermatologic expertise, and enhance data collection.	Limited internet access Lack of digital literacy
Shared international screening guidelines	↑ Standardization of research	Tailored protocols ensure consistency in research methodologies, improve the comparability of data, and aid in the development of targeted interventions.	Geopolitical instability Variability in health care infrastructure and regulation across countries
Advocacy for equitable research funding	↑ Continuity of research ↑ Availability of data	Advocacy efforts would help secure equitable global funding for research with vulnerable populations, strengthening the research process.	Entrenched funding inequities favoring institutions in high-income countries
Trauma-informed care training	↓ Psychological harm	Clinician education on trauma-informed care reduces emotional stress, enhances trust, and improves patient cooperation in research studies.	High clinician workload in humanitarian settings

Because of a frequent lack of access to primary care, displaced individuals often present urgently with dermatologic conditions, which can make management difficult and worsen the prognosis [2]. Delivering care close to the patient’s community through community-based models is one way to combat this deficiency, and specialist training of front-line health care providers (including the World Health Organization, Red Cross, the United Nations High Commissioner for Refugees, and Doctors Without Borders) may allow for earlier diagnosis and treatment [2].

For complex cases, teledermatology has emerged as a potential solution for the shortage of trained dermatologists working in this field [2,3]. In a population with high rates of communicable skin disease, teledermatology also limits the spread of infection [2,3]. Integration of systems to conduct medical care virtually would also address the lack of consistent access to hidden populations, enabling continuity of care regardless of the patient’s location [2,3]. Virtual platforms can facilitate improved understanding, confidentiality, and engagement with patient-centered, multimedia, interactive informed consent [17]. However, these platforms require stable internet access, compatible devices, and digital literacy for maximum effectiveness [17].

More research is needed to test the efficacy of standardized care models on dermatologic outcomes. Expanding the scope of these investigations requires the development of national screening guidelines for skin diseases in migrants and displaced persons, a task complicated by nation-specific differences in health care infrastructure and regulation (Table 1). To address the lack of resources, researchers should advocate for equitable global funding by raising awareness about the importance of research in vulnerable populations (Table 1). Clinicians looking to secure support can also form collaborative partnerships with agencies like the United Nations High Commissioner for Refugees and Doctors Without Borders.

Advancing dermatologic care for displaced populations requires an approach informed by ethical practices and cultural sensitivity. By addressing the unique challenges faced by displaced individuals, such as their legal uncertainties, high rates of infectious disease, and elevated potential for retraumatization, clinicians can work to develop more effective research strategies. Proposed solutions include advocacy for equitable research funding, development of uniform international screening protocols, use of teledermatology, and the integration of trauma-informed care into dermatologic services (Table 1). Further research is essential, and dermatologists must work with community health systems to craft and optimize care models.
